# Breakage of Needle during Intracavernosal Injection and Use of Portable Ultrasound Guidance for Removal

**DOI:** 10.1155/2013/215492

**Published:** 2013-06-02

**Authors:** Wayland Hsiao, Fei Lian, Brooks Goodgame, Chad W. M. Ritenour, Jordan Angell, Viraj A. Master

**Affiliations:** Department of Urology, Emory University School of Medicine, Atlanta, GA 30322, USA

## Abstract

*Purpose*. Intracavernosal self-injection (ICI) was first described in 1982, and remains a viable therapy for erectile dysfunction. However, intracorporal needle breakage can be a rare complication of therapy. We report a rare complication of intracorporal needle breakage and a retention of a 30-gauge needle in a 42-year-old paraplegic man. We discuss our experience in using portable high-frequency ultrasound intraoperatively to visualize and guide removal of a retained ICI needle. *Materials and Methods*. Review of case and ultrasound technique are presented. *Results*. Using intraoperative ultrasound imaging, the retained intracorporal needle was successfully removed from the patient's penis without any complications. Follow-up ultrasonography and X-ray confirmed complete removal of the needle. *Conclusions*. We report on the successful implementation and use of a portable high-frequency ultrasound probe to visualize a retained intracorporal needle inside the penis and its use to guide removal. Given the rapid proliferation of portable ultrasound machines in the operating room and out in the field, we expect these imaging techniques to become routine, especially in urological emergencies.

## 1. Introduction

Intracavernosal injection (ICI) therapy was introduced in 1982, and today it remains a viable second-line therapy for erectile dysfunction with high satisfaction reported in patients who remain on ICI [1, 2]. Complications of ICI may include penile burning, priapism, and ecchymosis [3]. Herein, we describe an uncommon complication of ICI therapy, namely, breakage of the ICI needle and lodgment of needle in the corpus cavernosum itself [4–6]. We further describe the novel use of bedside ultrasound visualization to localize the needle to guide removal. 

## 2. Materials and Methods

 The patient was a 42-year-old African-American male paraplegic with a history of transverse myelitis. He had been successfully treated with ICI for a number of years. While performing an injection early one morning, a large section of the 30-gauge needle broke at the hub, remaining lodged in the penis. The patient attempted to remove it himself at home by making a small incision over the injection site but was unable to do so. 

**Figure 1:**
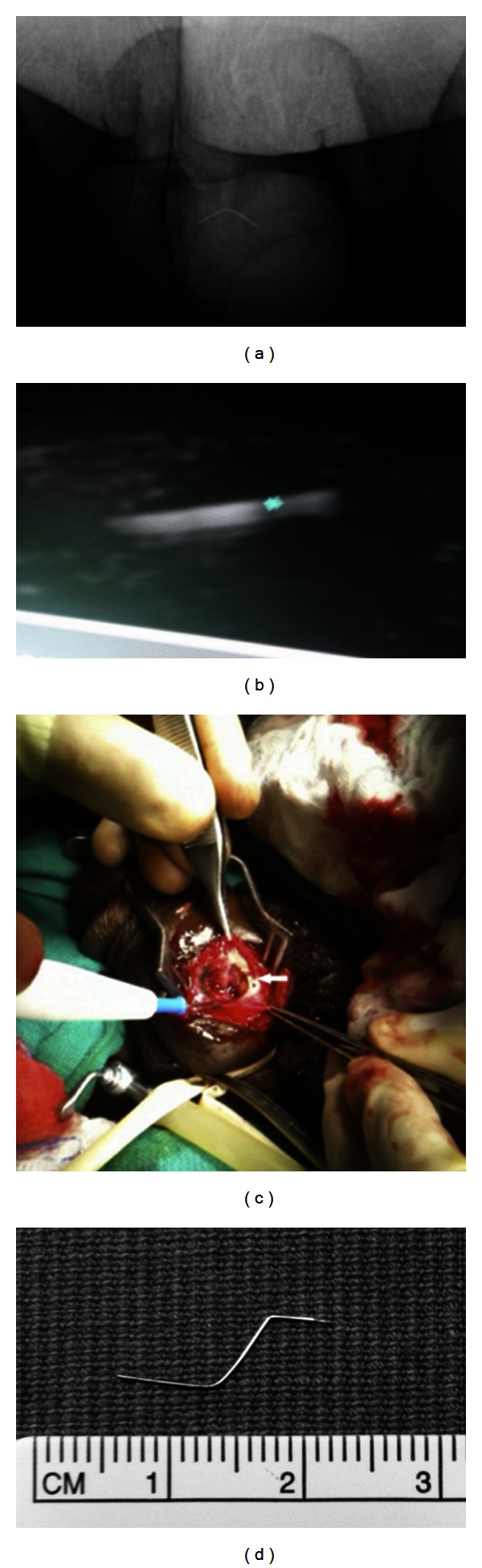
(a) Use of digital radiography to enlarge and increase brightness of the pelvic X-ray to better demonstrate presence of the retained needle. (b) Image of broken intracavernosal needle deep within penile tissue, via portable high-frequency ultrasound. (c) Intraoperative photograph of needle embedded within penile tissue; white arrow shows needle location. (d) The broken needle after extraction from the patient's penis.

 He presented to the Emergency Department without any gross hematuria. A pelvic X-ray revealed a 30-gauge needle in the penis (Figure 1(a)). On physical exam, the needle was not immediately palpable, although deep palpation was not attempted secondary to risk of needle-stick exposure. Other laboratory values were within normal limits.

 The patient was brought to the operating room for penile exploration and removal of foreign body. On flexible cystoscopy, no needle was visualized in either the bladder or the urethra. At this point, we obtained a portable high-frequency ultrasound probe for visualization. The machine used was the same that anesthesiologists use to place venous lines; thus, it is readily available in nearly all operating rooms. No specific settings were changed, as the default setting, 12 MHz, is readily used to detect structures at a short distance.

## 3. Results

 On ultrasound imaging, a hyperechoic longitudinal structure was seen, corresponding to the broken intracorporal needle (Figure 1(b)). A 1 cm longitudinal incision was made directly above where the needle was buried. The needle was extracted without complication (Figures 1(c)-1(d)). There were no operative complications. Follow-up ultrasonography and X-ray confirmed successful removal of the broken intracorporal needle in its entirety. 

## 4. Discussion and Conclusions

We report on a novel use of portable high-frequency ultrasound to visualize a foreign body inside the penis before surgical intervention. While needle break complications are rare, there are other reported cases of needle breakage and retrieval during ICI [6, 7].

The described operative technique represents intraoperative utilization of a portable high-frequency ultrasound probe to precisely locate a foreign body in the deep tissues of the penis. This allowed for rapid extraction of the needle with minimal injury to the patient, protection for the surgical team from potential needle-stick injury, and reduction of exposure to ionizing radiation by avoiding intraoperative fluoroscopy. Other urologists and surgeons may find this technique helpful for the removal of foreign bodies from deep tissues of the penis.
